# Open Heart Dual Valve Surgery Without Blood Transfusion: A Case Report

**DOI:** 10.7759/cureus.68875

**Published:** 2024-09-07

**Authors:** Anuj Timshina, Santosh S Parajuli, Sumnima Adhikary

**Affiliations:** 1 Anesthesiology, Shahid Gangalal National Heart Centre, Kathmandu, NPL

**Keywords:** blood transfusion, cardiac surgical procedures, cardiopulmonary bypass, jehovah's witness, rheumatic heart disease

## Abstract

In Nepal, rheumatic heart disease (RHD) is alarmingly prevalent, marked by presentations like migratory joint arthritis, carditis, subcutaneous nodules, erythema marginatum, and Sydenham chorea. This condition can progress to instigate valvular defects. Although these patients are first approached medically, they may require surgery for severe cases. Refusal for blood transfusion might not be a major issue for other general surgeries; however, in cardiac surgery, where there is massive blood loss, it’s quite a challenge. This challenge becomes even more pronounced in a developing country that lacks advanced facilities like a cell saver for autotransfusion. Herein, we report a case of a 22-year-old female, a Jehovah’s Witness, suffering from RHD, severe mitral regurgitation, severe tricuspid regurgitation, and severe pulmonary artery hypertension. She underwent mitral valve replacement and tricuspid repair surgery (modified DeVega) by avoiding any form of blood product transfusion.

## Introduction

Rheumatic fever is a multisystem disorder that typically presents with symptoms like fever, joint pain, lethargy, and loss of appetite around two to three weeks after a sore throat caused by the bacterium *Streptococcus pyogenes* (group A *Streptococcus*). The autoimmune nature of rheumatic fever leads to inflammation and damage to the heart valves, ultimately resulting in rheumatic heart disease (RHD). Globally, common valve disorders include RHD, aortic stenosis, mitral regurgitation, and aortic regurgitation, while aortic stenosis is more prevalent in developed countries [1]. A study on RHD estimated that in 2015, there were 33.4 million global cases, 10.5 million disability-adjusted life-years lost, and 319,400 deaths due to RHD [2]. Mortality from RHD is influenced by various factors, including an increased risk of atrial fibrillation, which can result in stroke, along with heart failure, pulmonary edema, and cardiorenal syndrome [3]. Although the global age-standardized mortality rate from RHD declined by 47.8% between 1990 and 2015, significant regional disparities persist [2]. The World Health Organization (WHO) and World Heart Federation have set a goal to reduce mortality from cardiovascular diseases, including RHD, by 25% by the year 2025 [4]. While medical management is the primary treatment approach, surgical interventions such as balloon valvuloplasty, valvotomy, or valve replacement may be required in cases where medical therapy fails or the disease is severe. Effective management involves early identification, thorough preoperative planning, meticulous intraoperative hemostasis, and preparation of alternative blood restoration techniques acceptable to the individual [5].

## Case presentation

**Figure 1:**
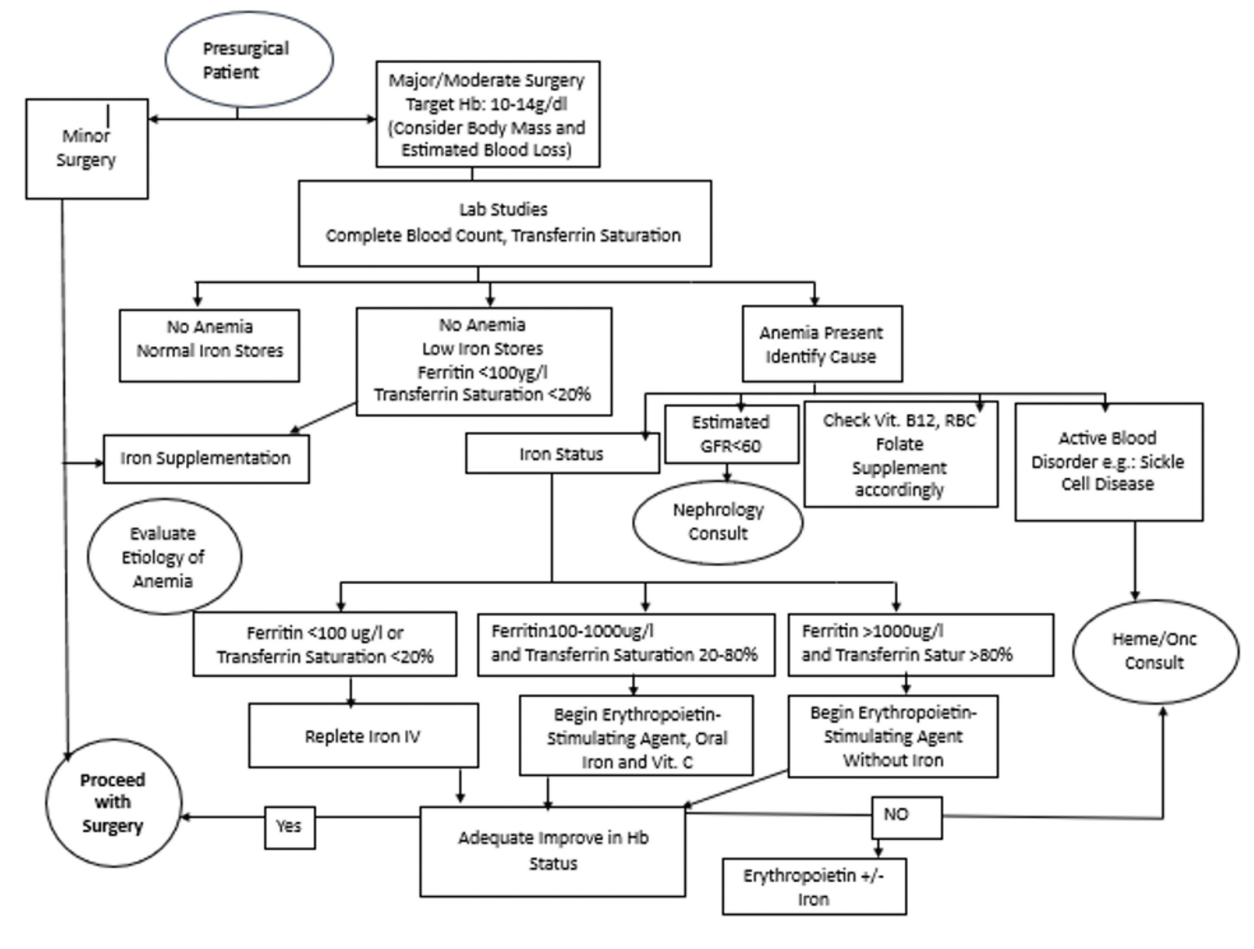
Algorithm for preoperative management of patients who are Jehovah’s Witness GFR: glomerular filtration rate; Vit: vitamin; RBC: red blood cell; Satur: saturation; Heme: hematology; Onc: oncology Adapted from Guinn NR, Resar L, Frank SM. Perioperative Management of Patients for Whom Transfusion Is Not an Option. Anesthesiology. 2021 Jun 1;134(6):939-48 [6]

A 22-year-old female with a five-year history of shortness of breath diagnosed with RHD presenting with severe mitral regurgitation and severe tricuspid regurgitation was referred to the Department of Surgery at Shahid Gangalal National Heart Centre, Kathmandu, Nepal, for surgical management. At her initial outpatient visit, she was evaluated, and her medication regimen of metoprolol 25 mg and furosemide 40 mg daily was noted. As a Jehovah’s Witness, she refused a blood transfusion, posing a challenge for the surgery. According to the algorithm in Figure 1 [6], she was evaluated for other comorbidities contributing to anemia that might necessitate a transfusion before surgery. Her vitamin B12, folate, and iron profiles were assessed, and all results were normal. Despite the algorithm's suggestion (Figure 1), she was started on oral iron supplements and scheduled for bi-weekly erythropoietin injections for four weeks prior to surgery. However, her non-compliance with erythropoietin and a positive SARS-CoV-2 status delayed the surgery twice. Upon final admission, she underwent examination and preoperative investigations. A repeat echocardiogram confirmed thickened mitral valve leaflets with severe mitral regurgitation, mild aortic regurgitation, severe tricuspid regurgitation, severe pulmonary arterial hypertension, and dilation of all cardiac chambers (left ventricular ejection fraction (LVEF): 50%). Her preoperative hemoglobin was 13.4 g/dL, her red blood cell (RBC) count was 4.6 million/μL, and her hematocrit was 40.9%. Other investigations were within normal limits.

**Figure 2:**
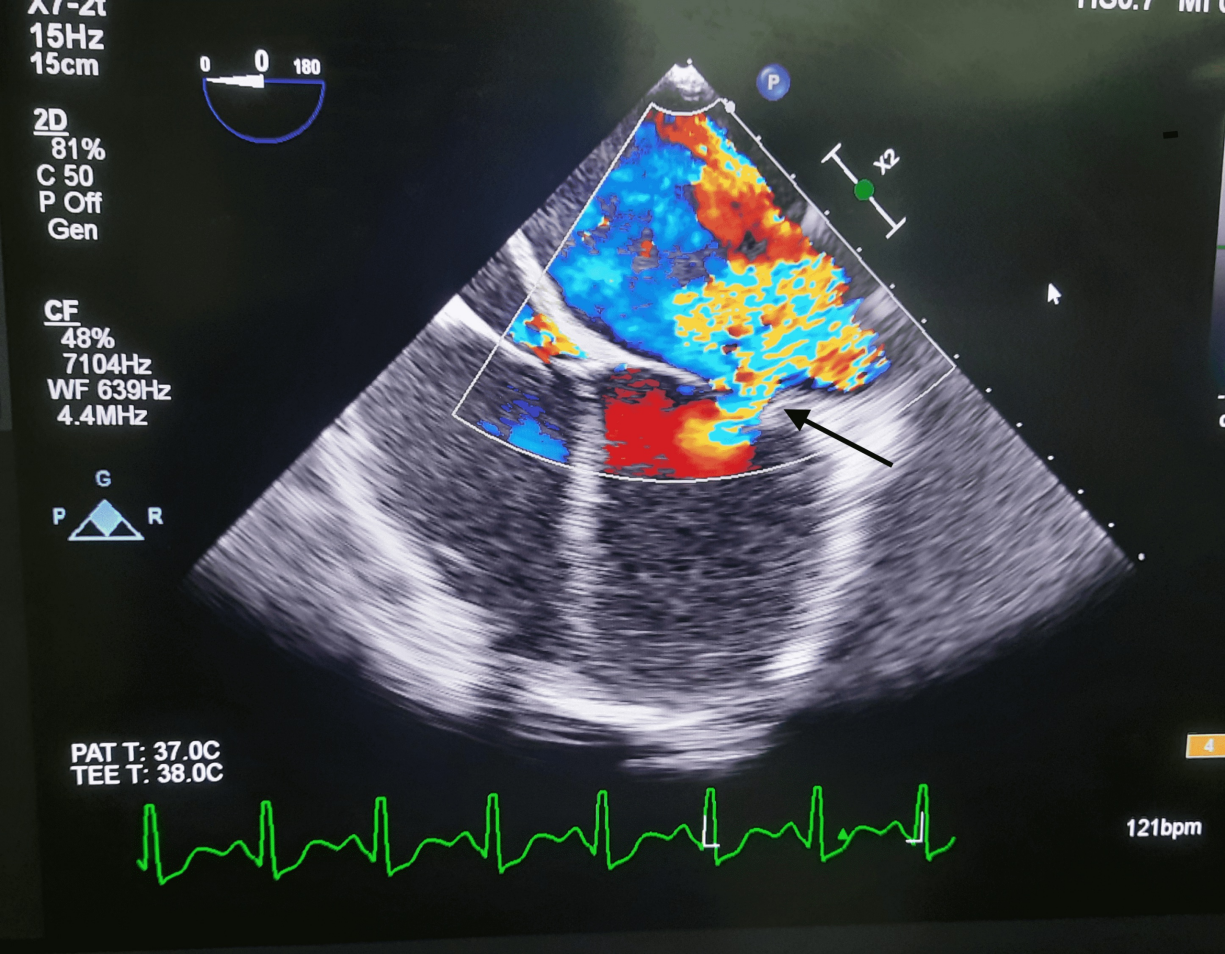
Severe mitral regurgitation with color jet viewed on transesophageal echocardiography

Intraoperatively, the patient was positioned supine and received peripheral, arterial, and central venous lines under aseptic conditions. She was started on minimal inotropic support with dopamine (2 mcg/kg/min), norepinephrine (0.01 mcg/kg/min), and continuous infusion of tranexamic acid. Anesthesia was induced with fentanyl, midazolam, propofol, and vecuronium, and endotracheal intubation was performed without difficulty. A transesophageal echocardiogram (TEE) was performed, revealing severe mitral regurgitation with a color jet, as illustrated in Figure 2. Following aseptic preparation and draping, a median sternotomy was performed. Cannulation of the aorta, superior vena cava, and inferior vena cava was done. Hemostasis was meticulously maintained at every step to minimize bleeding. Instead of the usual 1200 mL crystalloid solution for priming, retrograde autologous priming (RAP) was performed with 600 mL of the patient’s blood and 600 mL of plasmalyte. After establishing cardiopulmonary bypass (CPB), her hemoglobin dropped from 13.4 g/dL to 10.6 g/dL. The heart was arrested, and a right atriotomy was performed. The left atrium was accessed transeptally, and the mitral valve was excised and replaced. The intra-atrial septum was closed, and the tricuspid valve was repaired. Prior to weaning from CPB, modified ultrafiltration (MUF) was used to increase the hematocrit, raising her hemoglobin to 12.2 g/dL. Weaning from CPB was successful with continued inotropic support. Post CPB, her hemoglobin was 12.9 g/dL, and the CPB balance was negative 2650 mL. Decannulation was performed, and meticulous hemostasis ensured minimal blood loss, with an intraoperative drain volume of 200 mL. The chest was closed with sternal wires, and mediastinal drains were placed.

Postoperatively, oral iron and bi-weekly erythropoietin injections were continued. Routine postoperative management, including monitoring of arterial blood gases and hemoglobin levels, was performed. The postoperative drain volume was 150 mL over 24 hours. Although she required prolonged ICU care (five days) with inotropic support, her recovery was uneventful.

## Discussion

The Jehovah's Witnesses organization has over seven million members worldwide [5]. In Nepal, where 81.3% of the population is Hindu, the number of Jehovah's Witnesses is relatively small. Hence, performing high-risk surgeries such as cardiac surgery (dual valve replacement) without the option of blood transfusions is a challenge. In such cases, the first step is to educate the patient about the risks and document their refusal of transfusion. Following this, it is essential to plan an appropriate blood management strategy. This includes optimizing hemoglobin levels before surgery, minimizing blood loss and coagulopathy during the procedure, and addressing anemia and bleeding postoperatively [6]. Effective management involves early identification, thorough preoperative planning, meticulous intraoperative hemostasis, and preparation of alternative blood restoration techniques acceptable to the individual [5]. It is important to know that some patients who refuse major blood components like erythrocytes, platelets, and plasma may consent to minor fractions such as cryoprecipitate, albumin, immunoglobulins, and clotting factors [7-9]. Identifying these preferences well before surgery is crucial. Antifibrinolytics like aminocaproic acid and tranexamic acid enhance clot stability and are helpful in cardiac cases to reduce bleeding and the need for transfusions [10]. 

Several tools have been developed to assist in predicting the chance of undergoing blood transfusion in cardiac surgery, including the Transfusion Risk and Clinical Knowledge (TRACK) score, utilized by Kim et al., and the Transfusion Risk Understanding Scoring Tool (TRUST), used by Moraca et al. [11, 12]. A TRACK score cutoff of 22 out of 32 provided optimal sensitivity (67%) and specificity (73%) for predicting the need for perioperative transfusions [13]. These scores can be useful in evaluating morbidity and mortality in patients who refuse transfusion. Although most algorithms, like the one above, suggest only the use of iron and erythropoietin under failure to achieve target hemoglobin (10-14 gm/dl), our patient was started on oral iron and erythropoietin as a precautionary measure. After four weeks of premedication with oral iron tablets and erythropoietin injections, our patient's hemoglobin level raised to 13.4gm/dl. The efficacy of erythropoietin was specifically investigated by Duce et al. in a matched cohort study. This study result showed no clinically significant differences in outcomes between the two groups (patients treated with erythropoietin who declined blood transfusions to those who did not receive erythropoietin) [14].

Although patients may not consent to storing their own blood, such as through preoperative autologous donation, procedures involving autologous blood are generally accepted if the blood remains within a closed circuit, such as with blood cell salvage [15]. Therefore, our case was more challenging as advanced technologies like cell savers are not available. Some studies suggest RAP may reduce intraoperative and overall hospital red cell transfusions and mitigate hemodilution in cardiac patients [16]. However, Foreman et al. found no significant differences in key outcomes between groups with and without RAP, except for a small increase in postoperative ICU albumin infusion [17]. The clinical significance of RAP remains uncertain, and further research is needed. However, we employed the RAP technique for priming before initiating CPB to prevent hemodilution. Considering pros and cons, to enhance hematocrit concentration, MUF was done. Implementing these strategic measures resulted in minimal intraoperative bleeding, with a postoperative hemoglobin level of 12.9 g/dl.

A study at the Institute for Blood Management (2008-2021) found that 5.6% of patients had hemoglobin levels ≤8 g/dL, with lower levels linked to higher morbidity and mortality [18]. For each gram drop in hemoglobin, the risk increased 1.48 times. In the postoperative phase, the patient's recovery was facilitated by restarting oral iron supplementation and administering erythropoietin. To minimize blood loss, routine postoperative blood examinations were reduced. On the second postoperative day, the patient's hemoglobin level measured 10.6 g/dl. Despite a prolonged stay in the ICU due to the necessity of ionotropic support with dopamine and norepinephrine for four days, no other complications were encountered. Pattakos et al. found better long-term survival for patients who were Jehovah's Witnesses compared to non-Witnesses, and Jassar et al. reported favorable one- and five-year survival rates for Jehovah's Witness patients after cardiac surgery, suggesting that avoiding unnecessary blood transfusions can be beneficial [19, 20]. 

## Conclusions

Patients who refuse transfusions present significant challenges for high-risk surgeries, such as mitral valve replacement and tricuspid repair. Nevertheless, these procedures can be performed successfully with minimal risk when proper preoperative preparation and careful intraoperative management, including meticulous hemostasis, are applied. While various studies have demonstrated reduced morbidity, mortality, and length of hospital and ICU stays for patients without transfusions, highlighting the problems associated with unnecessary transfusions, our patient experienced a prolonged ICU stay. This underscores the importance of blood transfusion for better, faster, and smoother postoperative recovery. Despite these approaches, it is crucial to consider that in the event of intra- and postoperative complications, such as bleeding, the role of transfusion can be critical.
